# Prognostic role of N-Acetylgalactosaminyltransferase 10 in metastatic renal cell carcinoma

**DOI:** 10.18632/oncotarget.14786

**Published:** 2017-01-21

**Authors:** Li Liu, Ying Xiong, Wei Xi, Jiajun Wang, Yang Qu, Zhiyuan Lin, Xiang Chen, Jiaxi Yao, Jiejie Xu, Jianming Guo

**Affiliations:** ^1^ Department of Urology, Zhongshan Hospital, Fudan University, Shanghai 200032, China; ^2^ Department of Biochemistry and Molecular Biology, School of Basic Medical Sciences, Fudan University, Shanghai 200032, China

**Keywords:** metastatic renal cell carcinoma, GALNT10, prognosis, biomarker, tyrosine kinase inhibitors

## Abstract

**Background and Purpose:**

A previous study demonstrated that GALNT10 affects the sensitivity of cancer cells to tyrosine kinase inhibitor (TKI) therapy. The aim of this study was to assess whether GALNT10 holds a prognostic role in metastatic renal cell carcinoma (mRCC) patients treated with TKI agents.

**Results:**

GALNT10 had no statistical correlation with any other clinicopathological parameters except for route of gaining samples (*P* = 0.001) and Heng's risk stratification (*P* = 0.011). Patients with high level of GALNT10 had significantly shorter overall survival (OS) (*P* < 0.001) and progression-free survival (PFS) (*P* = 0.002). Importantly, this relationship existed in OS and PFS analyses in sunitinib-treated patients and in OS analyses in sorafenib-treated patients (*P* = 0.024). In contrast to sorafenib group, percentage of partial response (PR) and stable disease (SD) were higher in sunitinib group, while percentage of progression disease (PD) was much lower. Univariate and multivariate analyses identified that GALNT10 was an independent prognostic factor for OS (HR = 1.938, *P* = 0.014), not for PFS (HR = 1.532, *P* = 0.065), in mRCC. Incorporating it into Heng's risk model could sharpen its efficacy in distinguishing patients with potential higher risk.

**Materials and Methods:**

We retrospectively enrolled 138 mRCC patients treated with sunitinib or sorafenib at Zhongshan Hospital, Shanghai, China. A total of 111 valid cases were finally applied for analyses.

**Conclusions:**

These findings suggest that GALNT10 could be applied as a prognostic marker for OS in mRCC patients.

## INTRODUCTION

Renal cell carcinoma (RCC) accounts for 2–3% of malignancies in adults [1]. Although the use of modern abdominal imaging has led to an increase in localized RCC proportion at initial diagnosis [2], unexpected progression to metastasis often happens [3, 4]. Instead of traditional immunotherapy using IL-2 and IFN-γ for mRCC patients with limited benefit [5, 6], tyrosine kinase inhibitors (TKIs) therapy has shown significant survival extension of metastatic clear cell RCC (ccRCC) patients [7, 8]. Due to the various reactions to TKIs among patients, MSKCC and Heng's risk model are chronologically raised to stratify patients with different risks [9]. Due to the limitations of current risk models, researchers considered that adding molecular biomarker may be of help [10].

Aberrant glycosylation is common and representative in cancers including RCC [11]. They participate in malignant transformations and progression and many of them, such as carbohydrate antigens (CA)-125, CA-129, glycoprotein PSA, have been commonly applied as tumor markers in clinical practice. Most of the carbohydrate antigens are mucin-type O-linked glycans which were initiated by N-acetylgalactosaminyltransferases (GALNTs) [12]. So far twenty members of GALNTs have been identified, including GALNT1-14 and GALNTL1-6, and many are closely connected with malignancies [11, 12]. For example, GALNT3 expression level has been identified to be significantly associated with tumor behavior or prognosis in pancreas adenocarcinoma, renal cell carcinoma and gastric cancer [13–16]; GALNT6 is an independent indicator in mammary cancer [17]. GALNT10 stays low in normal renal tissue, but elevates in kidney cancer [18]. Patients with higher tumoral GALNT10 had a poorer OS and PFS in our previous study [18]. GALNT10 could enhance EGFR membrane retention via O-glycosylation. GALNT10 silencing increases sorafenib sensitivity of hepatoma cells [19]. EGFR also functions in renal cell carcinoma and is involved in the progression of renal cell carcinoma [20]. In this study, we sought to set a further step to assess whether GALNT10 holds a prognostic role in mRCC patients treated with TKI agents.

## RESULTS

### Characteristics and association with GALNT10 level

The baseline characteristics of this cohort were shown in Table 1. All patients were diagnosed with metastatic renal cell carcinoma. Most of the patients were male (71.2%) and clear cell subtype accounted for 89%. In Heng's risk stratification model, 60 cases (54.1%) were in the intermediate risk group, while 23 (20.7%) and 28 (25.2%) were classified into favorable risk group and poor risk group, respectively. GALNT10 level was separated into low and high level by median cut-off (Supplementary Figure 1). Association between baseline characteristics and GANLT10 level was exhibited in the same table. It is obvious that GANLT10 was associated with route of gaining samples (*P* = 0.001) and Heng's risk (*P* = 0.011). Other characteristics were not statistically associated with GANLT10.

**Table 1:** Clinical characteristics of patients according to GALNT10 expression

Characteristics	Patients		GALNT10 expression		
	*n*	%	low	high	*P*-value
All patients	111	100	55	56	
Age					0.297
≤ 59	56	50.5	25	31	
> 59	55	49.5	30	25	
Tumor size					0.804‡
≤ 4 cm	18	16.2	11	7	
> 4 and ≤ 7 cm	49	44.1	20	29	
> 7 and ≤ 10 cm	30	27.0	16	14	
> 10 cm	14	12.6	8	6	
Gender					0.952†
Female	32	28.8	16	16	
Male	79	71.2	39	40	
Prior nephrectomy					
Yes	111	100			
No	0	0			
Diagnosis					
Metastatic renal cell carcinoma	111	100			
Route of gaining samples					0.001†
Curative surgery	53	47.7	35	18	
Cytoreductive surgery	58	52.3	20	38	
Histology					0.063†
Clear-cell	89	80.2	48	41	
Non-clear cell	22	19.8	7	15	
Initial TNM stage					0.220†
I–III	52	46.8	27	25	
IV	59	53.2	28	31	
Fuhrman grade					0.395‡
1	2	1.8	2	0	
2	54	48.6	26	28	
3	41	36.9	21	20	
4	7	6.3	4	3	
Heng's risk model					0.011‡
Favorable risk	23	20.7	16	7	
Intermediate risk	60	54.1	31	29	
Poor risk	28	25.2	8	20	
Number of disease sites					0.727†
1	77	69.4	39	38	
≥ 2	34	30.6	16	18	
Sites of disease					
lung	83	74.8			
bone	18	16.2			
brain	2	1.8			
other sites	13	11.7			
Treatment					0.591†
sunitinib	74	66.7	38	36	
sorafenib	37	33.3	17	20	

^†^χ^2^ test or Fisher's exact test, ‡Cochran-Mantel-Haenszel χ^2^ test, *P*-value < 0.05 was regarded as statistically significant. Abbreviations: KPS, Karnofsky performance status.

### Impact of GANLT10 on survival of mRCC patients

**Figure 1:**
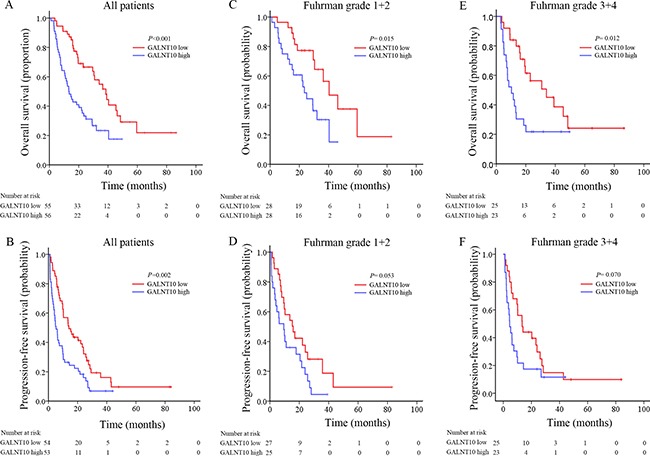
Kaplan-Meier analyses for prognosis of mRCC patients according to GALNT10 level (**A**) OS in all patients; (**B**) PFS in all patients; (**C**) OS in stratified low grade patients; (**D**) PFS in stratified low grade patients; (**E**) OS in stratified high grade patients; (**F**) PFS in stratified high grade patients.

Sixty-four percent of patients (71/111) died in this cohort. To assess whether GALNT10 was associated with clinical outcome, Kaplan-Meier analyses were done. As shown in Figure 1, GALNT10 was significantly correlated with OS and PFS (*P* < 0.001 and = 0.002, Figure 1A and 1B), in which high GALNT10 leveled patients experienced more death or disease progression. Meanwhile, high level of GANLT10 maintained its correlation with poorer OS both in low (Fuhrman 1 + 2, *P* = 0.015) and high (Fuhrman 3 + 4, *P* = 0.012) grade, but lost the significance in Fuhrman-based PFS subgroup analyses.

**Figure 2:**
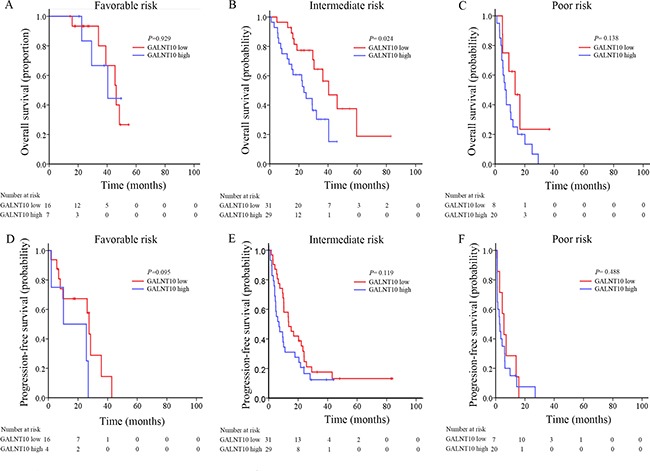
Kaplan-Meier analyses for prognosis of mRCC patients according to GALNT10 level in different Heng's risk groups (**A**) OS in favorable risk patients; (**B**) OS in intermediate risk patients; (**C**) OS in poor risk patients; (**D**) PFS in favorable risk patients; (**E**) PFS intermediate risk patients; (**F**) PFS in poor risk patients.

**Figure 3:**
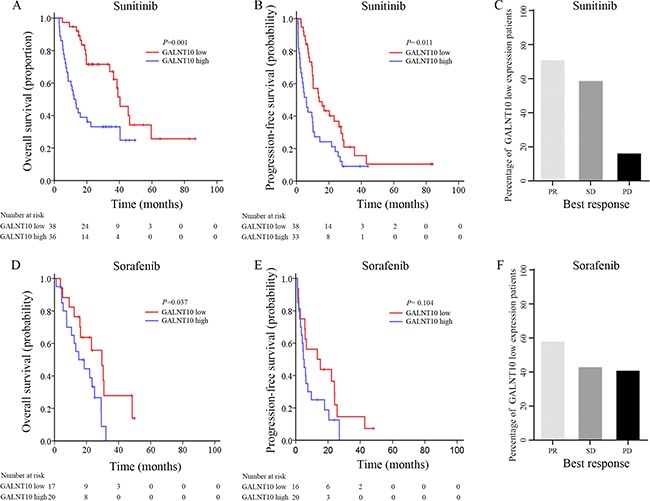
Survival and disease outcome after TKI therapy (**A**) OS analyses comparing high/low GALNT10 patients treated with sunitinib; (**B**) PFS analyses comparing high/low GALNT10 patients treated with sunitinib; (**C**) Percentage of response to sunitinib in low GALNT10 patients; (**D**) OS analyses comparing high/low GALNT10 patients treated with sorafenib; (**E**) PFS analyses comparing high/low GALNT10 patients treated with sorafenib; (**F**) Percentage of response to sorafenib in low GALNT10 patients.

Heng's risk stratification classified mRCC patients into three leveled groups. In this cohort, 20.7% (23/111), 54.1% (60/111) and 25.2% (28/111) of the cases were classified into favorable, intermediate and poor risk groups. GALNT10 exhibited its stratification ability only in intermediate risk patients in OS (*P* = 0.024, Figure 2). Interestingly and inspiringly, GALNT10 was significantly related with OS and PFS in patients treated with sunitinib (*P* = 0.001 and = 0.011, Figure 3A and 3B), and was only related with OS, not PFS in sorafenib group (*P* = 0.037 and = 0.104, Figure 3D and 3E). In GALNT10 low expressed patients, percentage of partial release (PR 70.0%) and stable disease (SD 57.9%) were higher in sunitinib group, compared with sorafenib (PR 57.1%, SD 42.1%), while percentage of progression disease (PD) was much lower in sunitinib group (15.4%) in contrast to sorafenib group (40%) Figure 3C, 3F. Supplementary Table 2 exhibited the distribution of best response in sunitinib and sorafenib subgroups, which indicated a much more significant discrimination in sunitinib-treated patients (*P* = 0.005). These data indicated that low GALNT10 level was potentially related with sunitinib response.

### Prognostic value of GANLT10 in mRCC

**Figure 4:**
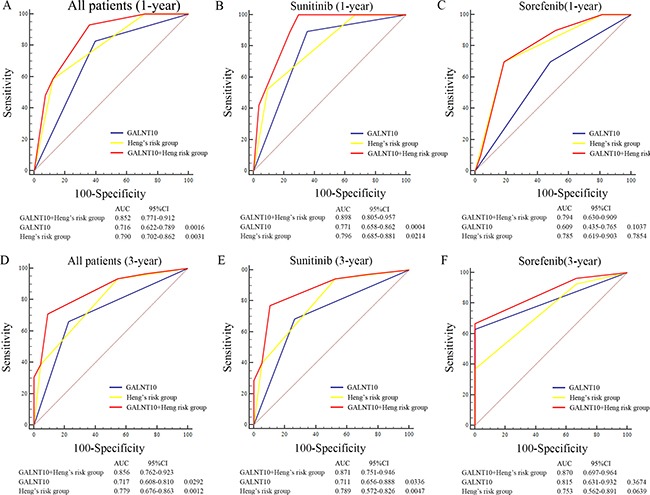
ROC analyses of predictive models in mRCC patients (**A**) ROC analyses in all patients at 1-year; (**B**) ROC analyses in patients treated with sunitinib at 1-year; (**C**) ROC analyses in patients treated with sorafenib at 1-year; (**D**) ROC analyses in all patients at 3-year; (**E**) ROC analyses in patients treated with sunitinib at 3-year; (**F**) ROC analyses in patients treated with sorafenib at 3-year.

To further determine the prognostic value of GALNT10, we applied univariate and multivariate Cox proportional hazard models to evaluate the HR and 95% CI. In univariate analyses, GANLT10 level together with histologic type, initial TNM stage, metastatic number and Heng risk group were associated with OS and PFS (Supplementary Table 1). In multivariate analyses, histologic type (HR = 2.395, *P* = 0.003), Heng's risk stratification (*P* < 0.001) and GALNT10 (HR =1.938, *P* = 0.014) were independent factors for OS. Interestingly, number of metastatic sites was independently associated with PFS (HR = 2.071, *P* = 0.002), while GALNT10 lost its significance, as *P* value was 0.065 Table 2. ROC analyses was further used to assess whether GALNT10 could improve current Heng's risk model. Exhibited in Figure 4, AUC of novel combined model was larger than Heng's risk model or GALNT10 alone both in 1-year and 3-year comparison (Figure 4A and 4D). TKI agents-based stratified comparison resulted in similar results. These findings demonstrated that GALNT10 was an independent prognostic factor for mRCC, and combining it with Heng's risk model sharpened the predictive efficacy.

**Table 2:** Proportional hazard model for overall survival and recurrence free survival prediction

Variables	OS (*n* = 111)		RFS(*n* = 106)	
	HR (95%CI)	*P*-value†	HR (95%CI)	*P*-value†
Histology				
Non-ccRCC vs ccRCC	2.395 (1.335–4.297)	0.003	1.700 (1.001–2.888)	0.050
Number of metastatic sites				
≥ 2 *vs* 1	1.534 (0.925–2.554)	0.097	2.071 (1.305–3.288)	0.002
Targeted therapy				
Sorafenib *vs* Sunitinib	1.296 (0.774–2.171)	0.324	1.328 (0.843–2.093)	0.221
Heng's risk group		< 0.001		0.005
Intermediate *vs* favorable risk group	2.163 (1.016–4.608)	0.045	1.211 (0.652–2.250)	0.544
Poor *vs* favorable risk group	6.755 (2.857–15.972)	< 0.001	2.707 (1.322–5.543)	0.006
GALNT10 expression				
High *vs* Low	1.938 (1.142–3.289)	0.014	1.532 (0.974–2.407)	0.065

ECOG PS = Eastern Cooperative Oncology Group performance status; HR = hazard ratio; CI = confidence interval; OS = overall survival; RFS = recurrence free survival; ^†^Data obtained from the Cox proportional hazards model, *P*-value < 0.05 was regarded as statistically significant.

## DISCUSSION

In this study, we sought to explore the prognostic role of GALNT10 in mRCC patients treated with TKIs. It is obvious that high level of GALNT10 was associated with shorter OS and PFS. Meanwhile, multivariate analyses revealed that GALNT10 level was an independent prognostic factor for OS, not PFS, of mRCC patients.

The polypeptide N-acetylgalactosaminyltransferases (GALNTs) family initiates mucin-type O-glycosylation [21]. It has been reported to be associated with malignancy in many studies. High level of GALNT3 was closely associated with poor disease-specific survival in renal cell carcinoma with a significant hazard ratio (HR = 3.43) [14]. GALNT10 as a member of GALNTs family was first reported in 2002 [22], but was little studied in the literature. In one previous study, Wu et al found it an adverse indicator for ccRCC [18]. In the present study, GALNT10 was more prevalent in Heng's high risk patients, and was not associated with other parameters. These findings might indicate a similar role of GALNT10 to Heng's risk stratification. Further multivariate analyses confirmed this hypothesis in mRCC (HR = 1.938, *P* = 0.014). Albeit limited researches, how GALNT10 affects malignancy could be conjectured by reviewing mechanisms of other GALNTs. Silencing GALNT7 could dramatically increase immunosuppressive cytokine interleukin-10, and subsequently result in T cell reduction [23]. GALNT7 expression could be regulated by many microRNAs, including miR-30b/30d, miR-34a/c, miR-494, miR-17-3p and so on [23–26]. Wu et al investigated GALNT10 in liver cancer and similar situations were found [19]. GALNT10 promotes proliferation and increases apoptosis resistance, and meanwhile is regulated by miR-122 [19]. Interestingly, hepatoma cells became more sensitive to sorafenib when GALNT10 was silenced [19]. This is consistent with the findings in this study that patients with low level of GALNT10 responded better to sunitinib or sorafenib.

TKI therapy was an emerging choice for doctors and patients. So far sorafenib and sunitinib are both commonly used for mRCC patients in China. However, similar patients treated with sunitinib or sorafenib may have quite distinct outcomes. Sunitinib was not available here in China a few years ago. As a result, some of the patients were treated with sorefenib instead of sunitinib. Thus separating patients with potential high risk for populations in the wild is an important task for urologists. GALNT10 is capable of distinguishing potential high-risk patients treated with TKIs, especially sunitinib-treated patients. Meanwhile, more PR to sunitinib in patients with low GALNT10 level could be observed compared with sorafenib. Therefore, GALNT10 tends to be more potentially effective in patients treated with sunitinib.

Being retrospective and small sample size are the major limitations of this study. One important reason is the relative short time period since TKI therapy was used in China. In addition, only sunitinib and sorafenib are involved in our study because other targeted agents were then not available. Finally, downstream of GANLT10 has not been fully interpreted, and how the downstream molecules affect cancer biology and the efficacy of TKI therapy needs to be further studied.

In conclusion, these findings suggest that GALNT10 could be applied as a prognostic marker for OS in mRCC patients.

## MATERIALS AND METHODS

### Patients

A total of 138 patients mRCC patients treated with TKIs (sunitinib or sofarenib) were enrolled between Mar 2005 and Jun 2014 at the Department of Urology, Zhongshan Hospital, Fudan University. Ethical approval was authorized by the Clinical Research Ethics Committee of Zhongshan Hospital, Fudan University (B2015-030). Informed consent was obtained from each patient. After adapted to inclusion and exclusion criteria, 111 patients were taken into analyses. Inclusion criteria were 1). diagnosis of mRCC, 2). treated with sunitinib or sorafenib as first-line systemic therapy, 3). no history of other malignancy and 4). with available fixed tumor tissues. Exclusion criteria were 1). former systemic therapy, 2). necrosis area > 80% in formalin-fixed, paraffin-embedded (FFPE) tissue blocks and 3). loss of follow-up. Baseline information, clinical and laboratory data, TKI therapy-related and survival data were collected from electronic medical records. Metastasis were diagnosed by imaging examination. Histology and nucleus grade were confirmed by a genitourinary pathologist. Tumor stage at operation was reclassified according to the 2010 AJCC TNM classification^20^. Progression definition followed the RECIST 1.1 criteria [27].

### Tissue microarray and immunohistochemistry

Tissue microarrays were manufactured as previously described [28]. The samples were obtained from either curative nephrectomy or cytoreductive nephrectomy. 1:600 dilated primary anti-GALNT10 antibody (Sigma-Aldrich, St Louis, MO, USA) was used in the IHC staining procedure. Operation process was performed as before [29]. Two random shots of each spot were obtained and all pictures were assessed in the semi-quantitative immunoreactivity score (IRS) system. IRS ranged from 0 to 30 which was the multiplication of intensity (0, negative; 1, weak; 2, intermediate; and 3, strong) and positive staining proportion (1 point for each 10% increment; the percentage of positive tumor cells ranged from 1 to 10). The average score of the pictures from one tumor represented the tumor IRS of the patient.

### Statistical

Overall survival (OS) and progression-free survival (PFS) were the primary outcomes. OS was defined as the time span from starting of TKI therapy to death of any cause. PFS was defined as the time span from starting of TKI therapy to progression date or last follow-up. GraphPad Prism 6 (GraphPad Software Inc., La Jolla, CA, USA) and SPSS 19.0 (SPSS Inc., IL, Chicago, USA) were used for process and evaluation of data. Median cut-off was done for high/low GALNT10 expression level. Connections between GALNT10 expression and clinicopathological characteristics were evaluated by χ2 test, Fisher's exact method or Cochran-Mantel-Haenszel χ2 test. Kaplan-Meier method and log-rank test were applied to determine the relationship between GALNT10 level and OS and PFS. Cox model based univariate and multivariate analyses were used to determine hazard ratio (HR) and 95% confidence interval (CI). The ROC analyses was performed to evaluate the efficacy of combinational prognostic models. Two tailed *P <* 0.05 was considered statistically significant.

## SUPPLEMENTARY MATERIALS FIGURES AND TABLES


