# Supplementary material for: Prognostic role of N-Acetylgalactosaminyltransferase 10 in metastatic renal cell carcinoma
Source: Oncotarget. 2017 Jan 21;8(9):14995–5003. doi: 10.18632/oncotarget.14786 (PMC5362461; doi:10.18632/oncotarget.14786)
Supplement: Supplementary file 1 [file oncotarget-08-14995-s001.pdf]

Prognostic role of N-Acetylgalactosaminyltransferase 10 in metastatic renal cell carcinoma

Supplementary Materials

Supplementary Table 1: Univariate analysis of characteristics associated with overall survival

Variables	Univariate analysis			Univariate analysis		
	Hazard Ratio	95%CI	*P*-value†	Hazard Ratio	95%CI	*P*-value†
Age, years	0.986	0.967–1.004	0.123	0.989	0.972–1.006	0.195
Gender						
Male *vs* Female	0.927	0.616–1.702	0.927	1.311	0.813–2.115	0.266
Histology						
non-ccRCC *vs* ccRCC	2.143	1.244–3.693	0.006	1.601	0.979–2.619	0.061
Fuhrman grade			0.377			0.834
2 *vs* 1	2.772	0.370–20.769	0.321	1.509	0.360–6.327	0.574
3 *vs* 1	3.815	0.512–28.426	0.191	1.587	0.378–6.661	0.528
4 *vs* 1	4.059	0.467–35.237	0.204	1.994	0.408–9.737	0.394
Heng's risk group			< 0.001			< 0.001
Intermediate *vs* favorable risk group	2.370	1.138–4.938	0.021	1.500	0.827–2.721	0.182
Poor *vs* favorable risk group	8.728	3.890–19.583	< 0.001	4.206	2.152–8.220	< 0.001
Treatment						
sorafenib vs sunitinib	1.616	0.996–2.620	0.052	1.360	0.882–2.097	0.164
GALNT10 expression						
High *vs* Low	2.492	1.528–4.065	< 0.001	1.919	1.266–2.908	0.002

KPS = Karnofsky performance status; LLN = lower limit of normal; ULN = upper limit of normal; CI = confidence interval; OS = overall survival; RFS= recurrence-free survival; †Data obtained from the Cox proportional hazards model, *P*-value < 0.05 was regarded as statistically significant.

Supplementary Table 2: Best response to targeted therapy according to GALNT10 expression

Best response	PR	SD	PD
GALNT10 low	18	30	6
GALNT10 high	9	27	17
P-value		0.005†	

*evaluated according to RECIST version 1.1; †$\chi 2$ test; PR = partial response, SD = stable disease, PD = progressive disease, RECIST = the Response Evaluation Criteria in Solid Tumors.

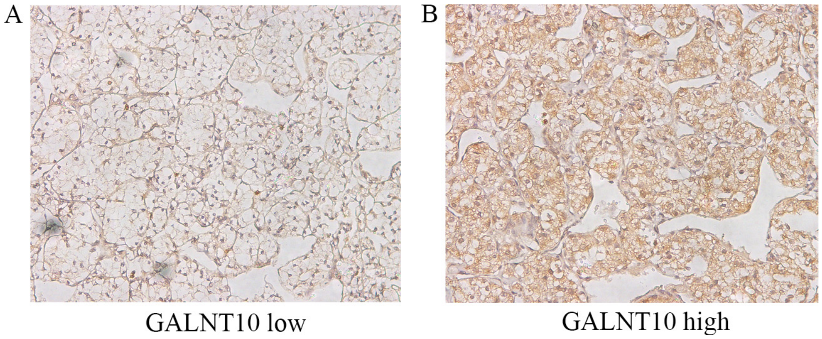

Supplementary Figure 1: Illustrative examples of GALNT10 immunohistochemistry staining. (**A**) low expression (IRS = 3); (**B**) high expression (IRS = 25).
